# From practice to perfection—complications and operative time learning curves in benign robotic-assisted laparoscopic hysterectomy

**DOI:** 10.1007/s11701-025-02429-8

**Published:** 2025-06-04

**Authors:** Klara Krantz Andersson, Anna Oksanen, Henrik Falconer, Malin Brunes

**Affiliations:** 1 Division of Obstetrics and Gynecology, Stockholm South General Hospital, Södersjukhuset, 118 83 Stockholm, Sweden; 2 Division of Biostatistics, Institute of Environmental Medicine, Karolinska Institutet, Stockholm, Sweden; 3 Department of Women’s and Children’s Health, Karolinska Institutet, Stockholm, Sweden; 4 Department of Clinical Science and Education, Karolinska Institutet, Södersjukhuset, Stockholm, Sweden

**Keywords:** Benign, Hysterectomy, Learning curve, Robotic-assisted surgery, Complications

## Abstract

This study aimed to estimate the learning curve for benign robotic-assisted laparoscopic hysterectomy, defined as the number of cases required to stabilize intra- and postoperative complications, estimated blood loss and operative time. This is a retrospective single-center cohort study with prospectively collected data. Patients who underwent a robotic-assisted laparoscopic hysterectomy between 2013 and 2021 were included. Six surgeons performed the surgeries. Analysis was conducted using linear and logistic generalized estimating equation (GEE) regression. The estimand was change in the speed of learning. The cohort comprised 1281 consecutive cases. An inverse association was observed between the number of robot-assisted laparoscopic hysterectomies and the number of complications with a breakpoint at 150 surgeries (adjusted Odds Ratio (aOR) 0.996, 95% Confidence Interval (CI) 0.992–0.999, *P* = 0.03) This decrease continued with surgeon experience. Moreover, there was a significant decrease in operative time after 50 operations (aCoeff -0.62 min, 95% CI − 0.89 to − 0.34 min, *P* < 0.001), with an operative time of approximately 100 min. No significant difference in intraoperative blood loss was observed throughout the learning curve (Knotbreak 50 operations, aCoeff 0.69 ml, 95% CI − 0.44 – 1.81 ml, *P* = 0.23). In this large observational study of learning curve for robotic-assisted laparoscopic hysterectomy, a plateau was reached at 50 cases for operative time and 150 cases for intra- and postoperative complications.

## Introduction

Surgical performance, often measured by operative time, complication rates or other metrics, typically improves with surgical experience. Graphically plotting performance against experience generates a learning curve (LC) [1]. An estimated LC is considered central when adopting new medical technology and essential when evaluating clinical outcomes, surgical metrics and cost–benefit analyses [2].

Robotic assisted surgery (RAS) is widely accepted owing to its technical advantages, but analyses of its implementation are lacking. Numbers of robotic surgeries in benign gynecology increases rapidly each year [3]. Because safety and accessibility are two of the main concerns in health care today, it is essential to assess this technology’s LCs, including both complications and operative time [2, 4]. Also, analyzing the LC of RAS is of importance from an individual and institutional perspective, as it may facilitate the development of surgical training programs and the implementation of new surgical centers [2].

The LC for robotic-assisted laparoscopic hysterectomy (RALH), often defined as reaching a plateau in operative time, is suggested to be between 20 and 91 cases [1, 5–10]. Earlier studies – primarily focused on operative time – have shown different and sometimes contradictory results and are limited by small data sets [1, 5–11]. Therefore, this study that include multiple active surgeons, a large number of RALH and the perspective of intra- and postoperative complications contribute with significant new empirical evidence on the LCs for RALH. This study aimed to estimate the LC defined as the number of cases required to stabilize the probability of intra- and postoperative complications and the operative time to perform a RALH. We also analyzed the LC for intraoperative bleeding.

## Materials and methods

This is an observational cohort study with prospectively collected data. Medical records of patients with benign gynecological disease who underwent robotic surgery between 1 November 2013 and 31 December 2021 at the Division of Obstetrics and Gynecology at Södersjukhuset in Stockholm, Sweden were reviewed. RAS for benign gynecology was introduced in 2013 at Södersjukhuset, Stockholm, Sweden. Since the start, over 2000 surgeries have been performed, the center is the most active in Sweden and one of the largest benign robotic centers in gynecology in Europe [12]. At Södersjukhuset, different surgical approaches are used for hysterectomy, and more complex cases are selectively assigned to robotic-assisted surgery (RAS). Patients with small uteri and signs of pelvic organ prolapse are typically managed with vaginal hysterectomy, whereas those with larger uteri, high BMI, suspected extensive adhesions, or severe endometriosis are preferentially selected for robotic-assisted procedures. All surgeries were performed by six gynecological surgeons using the da Vinci Surgical System Si/X (Intuitive Surgical, Sunnyvale, CA, USA). All surgeons were specialists in obstetrics and gynecology and had previous long experience in laparoscopic adnexal surgery and in abdominal hysterectomy but limited experience in conventional laparoscopic hysterectomy.

In the analysis of LCs only RALH was included. Sacrocolpopexy with concomitant RALH, was excluded. When admitted to the hospital, all patients agreed to be a part of a local quality register. The data included patient demographics (e.g., age, body mass index [BMI, calculated as weight in kilogram divided by height in meters squared]), parity and previous abdominal surgery. Preoperatively, all patients received one single dose of antibiotic prophylaxis. For all procedures a uterine manipulator was used. Postoperatively, the surgeons completed the registry with procedure type, intraoperative complications, estimated blood loss, conversion to open surgery, total operative time, robotic console and docking time. Total operative time was defined as the time from the first incision to skin closure including docking time. Blood loss was estimated by an anesthetist nurse based on blood collected in the theater room. In addition, postoperative information, including uterus weight, length of hospital stay and complications, were recorded. Uterine weight was measured immediately after uterine removal. Hospital stay was measured from admission to discharge. Intraoperative complications were defined using the Classification of Intraoperative Complications (CLASSIC) [13]. Postoperative complications were categorized according to the Clavien–Dindo (CD) system [14]. If the surgeon was uncertain about how a peri- or postoperative complication should be classified, the issue was raised within a group of six surgeons, who then made a collective decision. The group preferred to assign a higher classification to complications in cases of doubt. For instance, if a patient presented acutely and no abnormal findings were identified, the condition was still classified as CD 1, based on the assessment of prolonged pain. All cases of vaginal apex infections requiring drainage via ultrasound were classified as CD 3a. Complications, referred to in the analyses of LC, is a composite outcome of all complications intra- and postoperative (yes/no).

## Statistical analyses

Categorical variables were presented as absolute numbers (%) while continuous variables were presented as mean (standard deviation). To determine where the plateau of the LCs occurred the variables were first analyzed using a regression model with restricted cubic splines of four knots to allow for flexibility. These results were graphed for visual inspection, based on which the cut points for further analysis were visually determined. Based on these analyses, natural splines were used to capture the improvements and their changes in a more easily interpretable form. The parameter of interest was the interaction of the learning slope and the time period, which indicated the change in the speed of change at the cut point. The analysis was done in four (sub)populations: all, uterus < 300 g uterus > 300 g and individuals with endometriosis.

The primary outcome was complications. The secondary outcomes were operation time and blood loss. The outcomes were analyzed using linear regression for operation time and blood loss (continuous outcome) and logistic regression for dichotomous complications (yes/no). The outcome variables were then analyzed with uni- and multivariable regression adjusting for baseline characteristics (Table 2). We used a GEE models for linear and logistic regression. GEE was chosen to adjust for the intra-person-correlation inherent in repeated measurement designs. Analysis was done in Stata version 16.0. A *P* value < 0.05 was considered statistically significant.

The study was considered exempt from ethical review by the Swedish Ethical Review Authority. The application for ethical approval (reference number 2022-05795-01) was rejected on the grounds that the project does not involve the processing of sensitive personal data. Furthermore, the project is not of a nature that falls under the Act (2003:460) on the Ethical Review of Research Involving Humans.

## Results

**Fig. 1:**
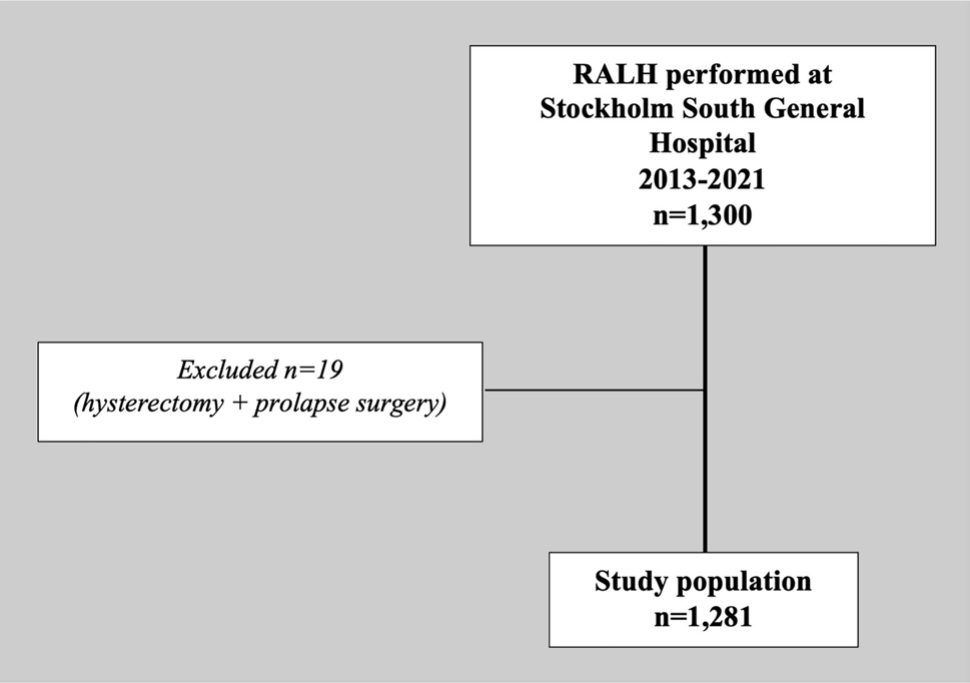
Flowchart of the study population. *n* = frequencies. Abbreviations: RALH = Robotic-assisted laparoscopic hysterectomy

One thousand seven hundred and ninety gynecological robotic-assisted surgeries were performed at Stockholm South General Hospital between 1 November 2013 and 31 December 2021. Of these 1,300 surgeries were RALH. 19 of these surgeries were excluded since they included both hysterectomy in combination with prolapse surgery (e.g., sacrocolpopexy). No data was missing. The final study population comprised 1,281 surgeries, as depicted in Fig. 1. Baseline characteristics and surgical data of the study population are summarized in Table 1.

**Table 1:** Summary of patient and operative characteristics

Patient characteristics *n* = 1281	Mean (± SD)	No. (%)	Surgical data *n* = 1281	Mean (± SD)	No. (%)
Age (years)	49.01 (± 9.22)		Operation time (min)	118.44 (± 61.47)	
BMI (kg/m2)	27.03 (± 5.65)		Blood loss (ml)	138.05 (± 205.69)	
ASA			Uterine weight (g)	354.22 (± 291.03)	
1–2		1133 (88.45)	Conversions		
> 3		144 (11.24)	No		1273 (99.38)
Missing		4 (0.31)	Yes		8 (0.62)
Parity (n)			Total complication (intra- or postoperative)		
0		284 (22.17)	No		1,043 (81.42)
> 1		996 (77.81)	Yes		238 (18.58)
Missing		1 (0.08)	Intraoperative complications		
Prior abdominal surgeries (n)			No		1250 (97.58)
No		636 (49.65)	Yes		31 (2.42)
Yes		645 (50.35)	Postoperative complications (CD)		
			0		1059 (82.67)
			1		66 (5.15)
			2		118 (9.21)
			3		38 (2.97)
			Length of stay (days)		
			0 (Day surgery)		174 (13.58)
			1		913 (71.27)
			> 1		194 (15.14)

Figures are mean values (standard deviation) or frequencies (proportions %)

Abbreviations: BMI = body mass index, ASA = American society of Anesthesiologists Physical Status Classification, CD = the Clavien–Dindo Classification of Surgical Complications

Complications (intra- or postoperatively) occurred in 238 (18.6%) cases. The most common intraoperative complication was ureter injury (*n* = 9, 0.7%), followed by bladder (*n* = 7, 0.5%) and bowel injury (*n* = 3, 0.2%). Postoperative complications included urinary tract infections (*n* = 37, 2.9%), local wound infections (*n* = 28, 2.2%) and—the most common one—vaginal cuff infections (*n* = 82, 6.4%). Reoperation—including ultrasound guided drainage of vaginal cuff infected hematoma—was necessary in 38 cases (i.e., CD3) and another 89 patients were readmitted mainly because of postoperative infections or postoperative hemorrhage not requiring a second surgery.

**Fig. 2:**
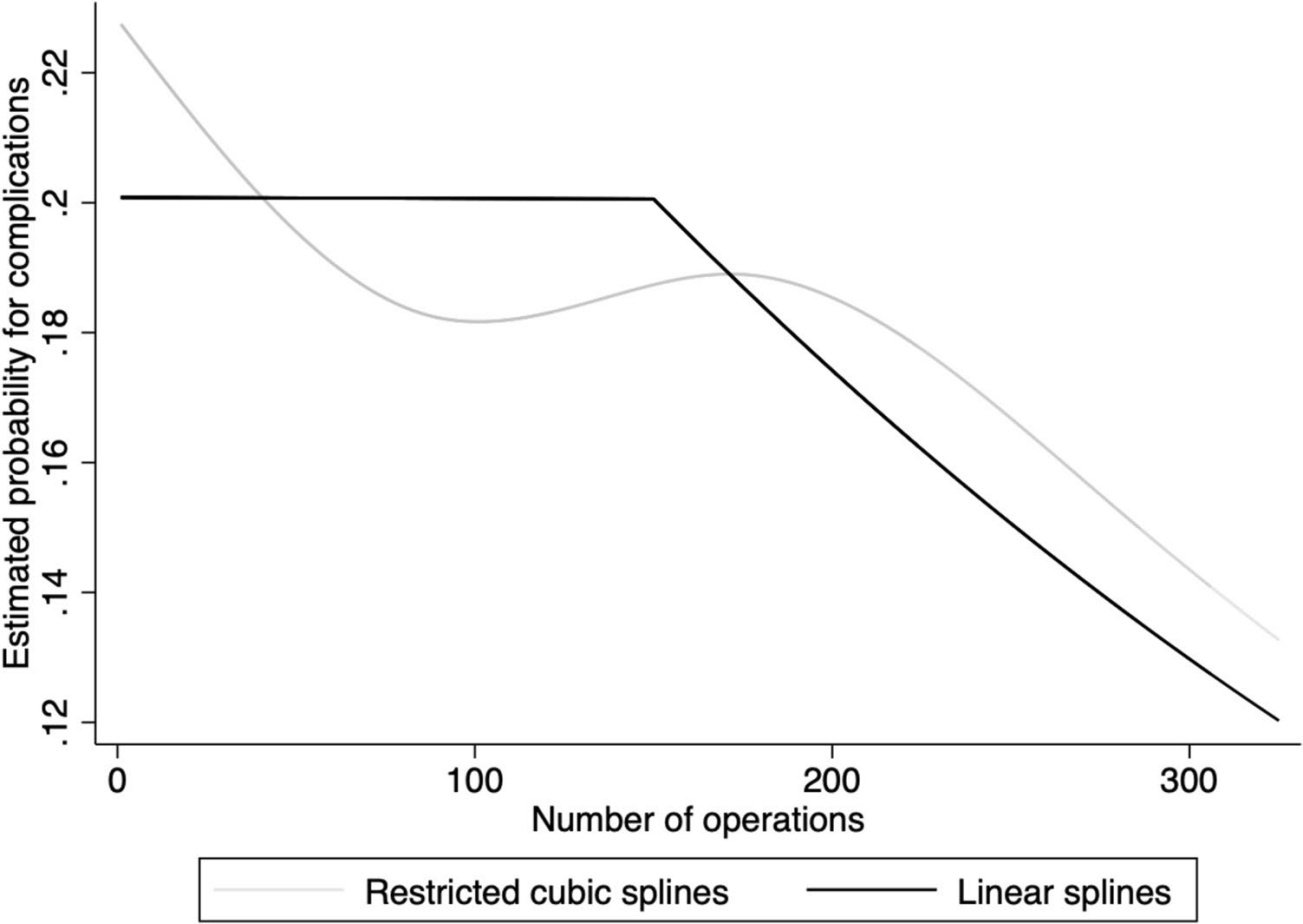
The rate of complications in relation to the number of operations performed. Estimated probability of intra- or postoperative complications in relation to the number of operations (i.e., RALHs) in restricted and linear splines

Figure 2 illustrates the estimated probability of intra- or postoperative complications to the number of RALHs performed, i.e., the LC for complications. There was a significant decline in total complications after 150 operations, and the graph also indicates that the decrease continued with surgeons’ experience.

**Fig. 3:**
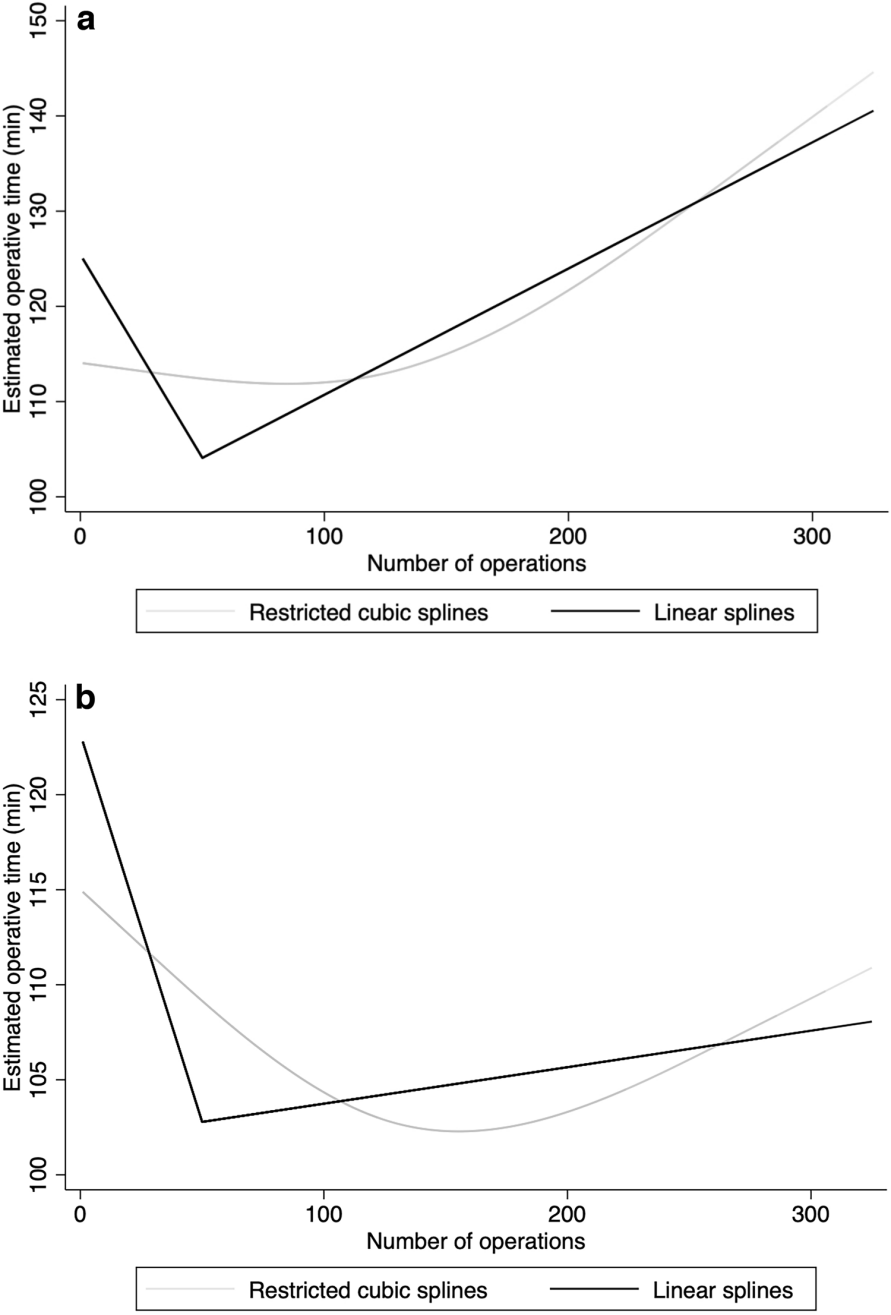
a Learning curve (operative time) for robotic-assisted laparoscopic hysterectomy including all patients. Estimated operative time (min) and number of operations in restricted and linear splines. b Learning curve (operative time) for robotic-assisted laparoscopic hysterectomy excluding patients with endometriosis. Estimated operative time (min) and number of operations in restricted and linear splines

Figure 3a displays the LC, defined as operative time, for all patients undergoing RALH and in Fig. 3b patients with endometriosis were excluded. A plateau was achieved after approximately 50 cases when reaching an operative time of about 100 min. In Fig. 3b, patients with endometriosis were excluded and in this subgroup the operative time remained consistently stable at about 100 min.

The results of the regression analyses are presented in Table 2. We found a significant difference in mean operative time after a knot break at 50 operations (aCoeff − 0.62 min, 95% CI − 0.89 to − 0.34 min, *P* < 0.001) (Fig. 3). This difference was also demonstrable between smaller (aCoeff − 0.51 min, 95% CI − 0.75 to − 0.26 min, *P* = 0.04) and larger (aCoeff − 0.53 min, 95% CI − 0.98 to − 0.09 min, *P* = 0.02) uteri, but not if the analysis only included patients with endometriosis (aCoeff 0.37 min, 95% CI − 2.58 – 1.8 min, *P* = 0.65). Intraoperative blood loss did not differ significantly after a knot break of 50 operations (aCoeff 0.69 ml, 95% CI -0.44 – 1.81 ml, *P* = 0.23), nor in total when subgrouping the operations by uterus size. Median blood loss was 75 ml. Finally, the adjusted regression analysis demonstrated a difference between the number of RALHs performed and the number of complications in which the knot was set to 150 operations (adjusted Odds Ratio 0.996, 95% Confidence Interval (CI) 0.992–0.999, *P* = 0.03). This difference was only significant when analyzing all the operations and not when subgrouping the patients according to uterus size or endometriosis.

**Table 2:** Regression analysis of operation time, blood loss, and complications of the knot breaks 50 and 150 operations, respectively

Outcome GEE^a^ linear regression	Knot break for number of operations	Estimate for 1st operation interval Coeff (CI)	*P* value	Estimate for 2nd operation interval Coeff (CI)	*P* value	Difference Coeff (CI)	*P* value
Operative time (min) Total	*50 operations*	− 0.64 (− 0.90 to −0.38)	< .001	− 0.02 (− 0.06 to 0.01)	0.22	− 0.62 (− 0.89 to − 0.34)	< .001
Uterus < 300 g^b^		− 0.53 (− 0.75 to − 0.31)	< .001	− 0.02 (− 0.07 to 0.02)	0.31	− 0.51 (− 0.75 to − 0.26)	0.04
Uterus > 300 g^b^		− 0.59 (− 1.00 to − 0.17)	0.05	− 0.05 (− 0.11 to 0.001)	0.06	− 0.53 (− 0.98 to − 0.09)	0.02
Endometriosis		− 0.20 (− 2.3 to 1.93)	0.86	0.17 (0.01 to 0.37)	0.74	0.37 (− 2.58 to 1.8)	0.65
Blood loss (ml) Total	*50 operations*	0.70 (− 0.34 to 1.74)	0.19	0.02 (− 0.15 to 0.18)	0.85	0.69 (− 0.44 to 1.81)	0.23
Uterus < 300 g^b^		0.34 (− 0.30 to 0.98)	0.30	− 0.25 (− 0.39 to − 0.11)	< .001	0.60 (− 0.12 to 1.31)	0.10
Uterus > 300 g^b^		0.46 (− 1.80 to 2.72)	0.69	− 0.17 (− 0.47 to 0.14)	0.28	0.63 (− 1.78 to 3.05)	0.61
Endometriosis		0.04 (− 6.39 to 6.46)	0.99	− 0.27 (− 0.76 to 0.21)	0.27	0.31 (− 6.35 to 6.97)	0.93

Outcome GEE^a^ logistic regression	Knot break for number of operations	Estimate for 1st operation interval OR (CI)	*P* value	Estimate for 2nd operation interval OR (CI)	*P* value	Difference OR (CI)	*P* value
Complications Total	*150 operations*	0.999 (0.995–1.002)	0.46	1.0 (0.997–1.009)	0.38	0.996 (0.992–0.999)	0.03
Uterus < 300 g^b^		1.001 (0.997–1.006)	0.58	1.007 (0.998–1.02)	0.13	0.994 (0.988–1.0)	0.06
Uterus > 300 g^b^		0.997 (0.991–1.003)	0.31	0.996 (0.99–1.01)	0.47	1.001 (0.995–1.007)	0.78
Endometriosis		1.001 (0.991–1.009)	0.90	1.01 (0.993–1.02)	0.31	0.993 (0.985–1.001)	0.07

^a^ Generalized Estimation Equations (GEE) models for linear and logistic regression

*Note* adjusted for BMI, parity, age, prior abdominal surgery, ASA classification, endometriosis (yes/no), specimen size

OR = Odds ratio, Coef = coefficient from linear regression model, CI = confidence interval

^b^ Patients with no endometriosis diagnosis

## Discussion

The main finding is the inverse association between the number of RALHs and the number of complications with a breakpoint at 150 performed surgeries, which provide a LC on complications for RALHs. The data also suggest that the decrease continues with surgical experience. For operative time, the LC for RALH stabilized after 50 cases with an operative time of approximately 100 min. The adjusted regression analysis showed that LCs remained stable when the surgical team engaged in more complex surgery (e.g., patients with larger uteri and advanced endometriosis).

Previous studies on the LC of RALH demonstrate a wide range in operative time [1, 5–7, 11] whereas none have addressed the potential impact on complications. This variation in operative time is likely a result of differences in study design and a limited number of analyzed cases. Also, in our study we could not identify a breakpoint with shorter operative time in endometriosis cases wich might be explained by a smaller subgroup and a rise in more complex cases during the study period. However, the operative time appears to remain stable when more complex procedures are introduced [1, 5–7, 9–11]. Our study confirms these findings. Moreover, it should be noted that the rate of severe complication (both intra- and postoperatively) was consistently low in our study (< 3%) (RALH with endometriosis was included), which is in line with the results of previous studies [15–17]. This low rate makes it challenging to identify significant differences. Regarding blood loss, our study did not observe any significant changes. This lack of a significant difference is likely because blood loss during RALH often is negligible, regardless of surgeon volume [18]. Other studies have reported a significant difference in blood loss associated with varying uterine sizes [19, 20]. In our study, LC did not affect difference in blood loss, regardless of uterine size.

While operative time is the most commonly reported LC metric in earlier RALH studies, it is crucial to question whether it is the best indicator of learning and proficiency. The discrepancy between reaching the plateau of the LC in operative time (50 performed surgeries) compared to the breakpoint in complication rates (150 surgeries) raises doubts about the adequacy of operative time as the primary metric for safe surgical training.

With the global establishment of robotic surgery in gynecology, the current results provide a basis for improved safety, efficiency and cost reduction associated with hospital and individual surgeon volume. In our study the complication rate decreased after 150 hysterectomies, suggesting that experienced surgeons should accompany novices until this plateau is reached. Naturally, surgical volumes of this size are a great challenge for both novices or more experienced surgeons in any gynecological center but may act as a target. We therefore further suggest that the surgical volume of gynecological departments and each individual surgeon should be centralized to create greater volumes per surgeon for maximum efficacy and safety. Finally, the suggested LC can be helpful in individual and institutional training programs and organizations, as well as to support and audit the introduction of RAS in gynecology without compromising patient safety.

The surgical LC is multifactorial, and current safety and training protocols remain limited. In a national survey onducted in Italy, 69.2% of surgeons reported using the operating room as the primary setting for acquiring laparoscopic skills [21]. A systematic review and meta-analysis evaluating the impact of three-dimensional (3D) versus two-dimensional (2D) imaging in laparoscopic training found a significantly higher error rate in the 2D group [22]. Future research on gynecologic RAS training may contribute to the development of evidence-based surgical education programs. The Society of European Robotic Gynaecological Surgery (SERGS) published a consensus document outlining recommendations for a standadised educational program in RAS [23]. With a stuctured curriculum and implementation of advanced simulation technique the learning process may be shortened in future.

Strengths of this study include the large number of consecutive cases performed by multiple surgeons and the completeness of the database, with almost no missing data. Furthermore, analyzing data on RALH only provides a more precise estimate of the LC for the most common major abdominal procedure in gynecology. In addition, single-center data limits the risk of substantial variations in the surgical technique that may affect the LC. However, generalizability of our results is uncertain as local tradition, structure and volume may have significant impact on the LC. Furthermore, the surgeons participating in this study had a long experience in laparoscopic adnexal surgery but only some experience within conventional laparoscopic hysterectomy.

As the current study only analyzed the impact of RALH on the LCs, extrapolation of these data to other robotic procedures should be made with caution. Finally, Södersjukhuset, Stockholm is one of four centralized hospitals for endometriosis care in Sweden and overrepresentation of patients with endometriosis may have influenced the results.

## Conclusion

Regarding RALH, a significant decline in complications was achieved after 150 cases, and the decrease continued with surgical experience. The LC for operative time was similar to previous data and no difference was observed in intraoperative blood loss throughout the LC. With the rapid implementation of robotic-assisted surgery worldwide, these results provide a basis for improved training curricula and increased centralization.

## Data Availability

We acknowledge the importance of data sharing for scientific transparency and reproducibility. However, our dataset contains coded register data from human participants. There is a key code at the register holders that can be used to trace back the data to living individuals. The existence of the code means that the data are “pseudonymized personal data” as per GDPR and cannot be published/shared openly. The European GDPR law on data protection and privacy imposes restrictions on openly sharing personal data. Our study has also been conducted in compliance with ethical guidelines, and the ethical review board has not authorized that the data will be made fully public. Sharing the data openly would not only constitute a GDPR breach but also a breach of the Swedish Ethical Approval law that has approved the research on the terms that personal data are protected as per GDPR and as per the conditions that are set by the register holders. For inquiries regarding access to the data, we would like to provide contact information for our Research Data Office at Karolinska Institutet: rdo@ki.se. They will handle requests for data access and ensure compliance with legal and ethical standards. In light of the restrictions outlined above, we propose the following Data Availability Statement: "The data in the study are pseudonymized (coded) personal data, and GDPR prohibits us from sharing this completely open. Data is available upon request, and requests for access to the data can be put to our Research Data Office (rdo@ki.se) at Karolinska Institutet."

## Abbreviations

a: Adjusted
BMI: Body mass index
CI: Confidence interval
CLASSIC: Classification of Intraoperative Complications
CD: Clavien–Dindo system
CLS: Conventional laparoscopic surgery
GEE: Generalized estimating equations
LC: Learning curve
OR: Odds ratio
RALH: Robotic-assisted laparoscopic hysterectomy
RAS: Robotic-assisted surgery
